# Barriers and facilitators to pharmacists’ engagement in response to domestic violence: a qualitative interview study informed by the capability-opportunity-motivation-behaviour model

**DOI:** 10.1093/pubmed/fdab375

**Published:** 2021-11-22

**Authors:** Natalia V Lewis, Tracey Stone, Gene S Feder, Jeremy Horwood

**Affiliations:** Centre for Academic Primary Care, Bristol Medical School, University of Bristol, Bristol BS8 2PS, UK; Institute of Population Health Sciences, Queen Mary University of London, London E1 2AB, UK; Centre for Academic Primary Care, Bristol Medical School, University of Bristol, Bristol BS8 2PS, UK; National Institute for Health Research (NIHR) Applied Research Collaboration West (NIHR ARC West) at University Hospitals Bristol NHS Foundation Trust, Bristol BS1 2NT, UK; Centre for Academic Primary Care, Bristol Medical School, University of Bristol, Bristol BS8 2PS, UK; Centre for Academic Primary Care, Bristol Medical School, University of Bristol, Bristol BS8 2PS, UK; National Institute for Health Research (NIHR) Applied Research Collaboration West (NIHR ARC West) at University Hospitals Bristol NHS Foundation Trust, Bristol BS1 2NT, UK

**Keywords:** health services, public health, violence

## Abstract

**Background:**

Domestic and sexual violence and abuse (DSVA) is a global public health problem resulting in health inequalities. Community pharmacies are uniquely placed to help people affected by DSVA. We examined factors that impact pharmacists’ engagement in response to DSVA when providing public health services.

**Methods:**

Semi-structured qualitative interviews with community pharmacists (*n* = 20) were analyzed thematically, with inductive themes mapped to the Capability–Opportunity–Motivation Behaviour (COM-B) model.

**Results:**

Pharmacists were confident in providing public health services, but a lack of DSVA training meant there is a need to support their ‘Capability’ to respond to DSVA. Pharmacies were perceived as highly accessible healthcare providers on the high street, with sexual health consultations offering an ideal ‘Opportunity’ to enquire about DSVA in a private consultation room. Pharmacist’s ‘Motivation’ to enquire about DSVA was driven by potential positive client outcomes and a desire to be more involved in public heath interventions, but organisation- and system-level support and remuneration is needed.

**Conclusions:**

Community pharmacy offers opportunities for integrating DSVA work in existing public health services. Pharmacists need training on DSVA, ongoing support, allocated funding for DSVA work, and awareness raising campaign for the public on their extended public health role.

## Background

Domestic and sexual violence and abuse (DSVA) is a global public health and clinical problem rooted in gender and social inequalities.^1^ In 2018, UK prevalence was 7.9% for women and 4.2% for men; lifetime prevalence was 29 and 13%, respectively.^2^ DSVA cost the UK economy £14 billion, £2.3 billion to health services and £1.3 billion to police.^3^ DSVA is a major source of health inequality,^4^ with related morbidity and mortality highest among women. The biggest impact is on mental, sexual and reproductive health.^5^ Consequences include sexual risk taking, reduction in contraception use,^6–8^ increased sexually transmitted infections (STI), unplanned pregnancy and abortion.^9^ The COVID-19 pandemic exacerbated gender and social inequalities,^10^ resulted in more DSVA,^11^ and increased demand for specialist DSVA services.^12^ The health-systems response to DSVA became more crucial than ever.^13^

Public Health England identifies community pharmacies as an important provider of public health services, uniquely placed to reach disadvantaged, marginalized and vulnerable populations.^14^ This role is clearly defined within the Healthy Living Pharmacy framework that aims to provide health promotion interventions tailored to local need, improve the health and wellbeing of the local population and reduce health inequalities. All UK community pharmacies must become Healthy Living Pharmacies in 2020–21.^15^ Pharmacies can play a crucial role for people affected by DSVA as a point of contact with healthcare professionals due to their high volume of daily contact with the public and high accessibility. Pharmacy as an accessible safe space for vulnerable populations became even more important during the COVID-19 pandemic. In 2020, the UK Home Office launched two codeword schemes (Ask for ANI, Safe Spaces) that allow DSVA victims to walk in a public establishment including pharmacy and ask for help with accessing police and specialist DSVA agencies.^16^ Some community pharmacies signed up for the schemes that provide training and promotional materials.^17^ Our scoping literature review found the gap in evidence for DSVA interventions in community pharmacies. The recent codeword schemes have not yet been formally evaluated.

World Health Organisation (WHO),^18^ National Institute for Health and Care Excellence (NICE),^19^ and the Department of Health and Social Care^20^ recommend DSVA training for healthcare professionals enhanced by system level support and multisectoral working.^19^ Systematic reviews provide strong evidence that awareness raising or training in isolation do not create consistent and sustainable change in professional behaviour.^21^^,^^22^ The evidence-based response to DSVA requires a comprehensive health-systems approach comprising: (i) capacity strengthening through repeated training and supervision for staff, (ii) leadership and political support, (iii) protocols to guide service delivery, (iv) coordination within the health system and across sector, (v) information systems for documentation and data collection, (vi) budgetary allocations, and (vii) infrastructure that allows for privacy and confidentiality.^23^ Identification and Referral to Improve Safety is an evidence-based system-level training, support and referral programme for behaviour change in general practitioner (GP) consultations with patients presenting with signs of DSVA (IRIS GP).^24^ In ten years, IRIS programmes have fully trained more than 1000 general practices and received referrals for 20 544 women in 48 localities across the UK.^25^ IRIS GP has been adapted for sexual health services.^26^

Our systematic review^27^ and study of a UK general practice dataset^28^ suggested that a request for emergency hormonal contraceptives (EHC) can help identify women who have experienced DSVA, and therefore, a consultation for EHC is an appropriate context for clinical enquiry and response to DSVA. In the UK, pharmacies supply up to 50% of all EHC.^29^ Women can obtain EHC via: (i) prescription from a healthcare practitioner, (ii) purchase over the counter for up to £35, (iii) free EHC for age group 13–24 commissioned by local authority under the Patient Group Direction (PGD). The PGD is a UK legal framework that allows registered pharmacists to supply medicine (e.g. EHC) to pre-defined groups of clients, without a prescription. The service is commissioned by public health departments in local authorities. The local authority pays pharmacies for each EHC PGD consultation and medicine.

A few studies explored the topic of DSVA in the community pharmacy setting. One US survey found that although women considered DSVA as an important health-care issue, they did not feel that a pharmacy would be an appropriate place for addressing this problem.^30^ The reasons overlapped with barriers found in other healthcare settings including issues around safety, timing and space.^31^ Two US surveys with pharmacy professionals^32^^,^^33^ identified additional barriers such as lack of skills, confidence and DSVA training. From communication with DSVA stakeholders, we learnt that in 2016 a specialist DSVA agency delivered IRIS GP training to a group of community pharmacists in London. This one-off training did not result in behaviour change; none referred clients experiencing DSVA to specialist agencies. This study formed part of the proof-of-concept research^27^^,^^28^ informing adaptation of IRIS GP for behaviour change in pharmacists health consultations. We aimed to understand the barriers and facilitators that impact pharmacists’ engagement in DSVA work when providing public health services.

## Methods

In March–July 2017, experienced female social science researchers (NVL, TS) conducted semi-structured interviews with community pharmacists purposefully sampled across two UK localities. We aimed to recruit a maximum variation sample for heterogeneity^34^^,^^35^ based on pharmacy characteristics and exposure to DSVA training. Local Pharmaceutical Committees (LPCs) in London and the South West of England provided contact details of pharmacies supplying more than five EHC a week under PGD. Consultation for EHC was chosen as an exemplar commissioned sexual health service provided by community pharmacies. TS sampled South West pharmacies where IRIS GP training had not taken place. She approached diverse pharmacies in relation to business type (standalone independent and corporate chains), size and area deprivation level of their location (measured with index of multiple deprivation (IMD) for English locations).^36^ LPCs emailed study information and invites to sampled pharmacies. In London, NVL approached all pharmacies that received IRIS GP training in 2016. Interested pharmacists contacted the researchers who arranged an interview at a mutually convenient time and place. Participants were offered a £20 shopping voucher.

Researchers introduced themselves and the study and obtained written informed consent. Interviews were guided by a topic guide developed with a pharmacist and LPC managers and pilot tested in the first two interviews. Interviews were audio-recorded, professionally transcribed and imported into NVivo 10 for coding. We used a combination of inductive thematic analysis^37^ and deductive mapping of the themes on the constructs of the Capability–Opportunity–Motivation Behaviour (COM-B) model.^38^ The COM-B model provides a framework to understand behaviour, drawing attention to the need to consider pharmacy staff ‘Capabilities’ and ‘Motivation’ to deliver the intervention, as well as contextual local ‘Opportunities’ in planning to introduce the intervention. NVL and TS started analysis of individual transcripts with open coding grounded in the data with JH double coding data in the first two transcripts. Differences in interpreting findings were discussed and agreed by NVL, TS and JH. Data were reviewed throughout the collection period at team briefings until we were satisfied that we had reached data saturation.^39^ NVL and TS wrote descriptive accounts of the inductive themes. NVL and JH mapped relevant themes on the COM-B constructs^38^ highlighting barriers and facilitators of pharmacists’ engagement in identifying and responding to DSVA when providing public health services.

## Results

LPCs emailed study invites to 35 pharmacies (24 in South West, 11 in London); 12 (9 South West, 3 London) did not respond, 3 (all London) declined due to workload; 20 pharmacists (15 South West, 5 London) consented to take part. Interviews (19 face-to-face in pharmacies, 1 telephone) lasted between 18 and 71 minutes (mean 33). We met our recruitment target on heterogeneity. The South West pharmacies representing independent and chain businesses were located in relatively deprived areas (mean IMD 3.6) across four local authorities. Included pharmacies served communities across both inner city and urban residential areas. Inner city pharmacies had lower IMDs, while urban residential settings were relatively less deprived. All London pharmacies were independent and located in less deprived residential areas (mean IMD 4.8) in one local authority (Table 1).

**Table 1:** Sample characteristics

Pharmacist/pharmacy	South Wes, *n* = 15	London, *n* = 5	Total, *n* = 20
Gender			
Female	8	2	10
Male	7	3	10
Years of practice mean (SD; range)	11 (10; 2–35)	19 (12; 5–35)	13 (11; 2–35)
Location of pharmacy			
Inner city	8	0	8
Urban residential	7	5	12
IMD of pharmacy area, decile mean (SD; range)	3.6 (2.1; 1–8)	4.8 (2.0; 2–7)	3.9 (2.2; 1–8)
Pharmacy business type			
Standalone independent	6	5	11
Corporate chain	9	0	9
DSVA training	2	5 (IRIS GP in 2016)	7

Note. DSVA—domestic and sexual violence and abuse. IRIS GP—Identification and Referral to Improve Safety in general practice. SD—standard deviation. IMD - the Index of Multiple Deprivation Deciles 2015 is calculated by ranking the 32 844 neighbourhoods in England from most deprived to least deprived and dividing them into 10 equal groups (1 most deprived, 10 least deprived).^36^

All pharmacies provided locally commissioned public health services other than EHC: chlamydia treatment (*n* = 10), supervised methadone consumption (*n* = 9), smoking cessation (*n* = 7) and flu vaccine (*n* = 7). All pharmacists had completed mandatory training on each public health service they provided and on safeguarding children and vulnerable adults. Only two pharmacists recalled encounters with a small number of clients who have experienced DSVA. We developed eleven themes from open coding and mapped them on the COM-B constructs. There was commonality in views across inner city and urban residential pharmacies.

### Pharmacists’ capacity to engage in identification and response to DSVA (Capability)

Pharmacists were confident in providing public health services, but a lack of DSVA training meant there is a need to support their ‘Capability’ to identify and respond to DSVA. All pharmacists felt adequately equipped for providing public health services. They perceived LPCs as trusted providers of training and advocates of pharmacists’ interests. All responders felt confident about having the necessary communication skills for approaching and responding to clients about health concerns, signposting to public health resources and using the safeguarding pathway. All pharmacists were committed to providing public health services, although everyone acknowledged tensions around multitasking and competing priorities (Table 2, quote 1). In contrast, only a minority of pharmacists said that they felt prepared to deal with clients affected by DSVA. Most participants reported lacking skills and confidence on how to ask about DSVA and respond to disclosure (Table 2, quote 2). Most pharmacists from South West struggled to recall DSVA training, policy/guidance and specialist services for referring/signposting affected clients. Some respondents expressed stereotypical ideas about signs of DSVA as mainly physical injuries. Pharmacists’ attitudes towards women who have experienced DSVA ranged from sympathetic to ambivalent and judgemental (Table 2, quote 3). All London pharmacists acknowledged that IRIS GP training increased their awareness and knowledge about DSVA. However, since 2016, only one pharmacist recalled one self-disclosure of DSVA. He offered a referral to the local DSVA agency, which the client declined. London pharmacists thought that they did not have opportunities and were lacking motivation for integrating extra DSVA work into existing public health services.

**Table 2:** Pharmacists’ Capacity to engage in identification and response to domestic and sexual violence and abuse (capability)

Quote number	Supporting quote	Participant
Quote 1	‘I’m very happy to do it [public health service]. What we find is that the time of the pharmacist! Well, we are providing the service, and there may be a queue of things happening outside. So sometimes I feel that I’m not able to spend as much time as I would like to, and it sometimes gets a bit rushed. Because subconsciously I’m thinking what’s going on outside. But that’s the only downside that I see. But on the whole providing a service is good, ‘cause we get good feedback from the patient.’	Pharmacist 1, standalone independent pharmacy, London
Quote 2	*‘it’s [domestic and sexual violence and abuse] a sensitive issue, and many people, and it’s then like we are busy like in the pharmacy. Sometimes we might miss something and it is more people to be aware of the signs I’d say as well um and there be pharmacists be more confident in order to, if they see something to actually be like maybe I should speak to someone about it, not report it directly but maybe speak to the safeguarding team of the area or speak to the GP if they think there is an issue, but then that is a bit difficult I’d say.’*	Pharmacist 2, corporate chain pharmacy, South West
Quote 3	*Researcher: ‘So, how do you feel about having those conversations [about domestic and sexual violence and abuse] with women?’ Pharmacist: ‘A bit out of my element…. Because obviously you want to help. You want them to… but then some of them sometimes go back [to their abusive partner].’*	Pharmacist 3, corporate chain pharmacy, South West

### External factors influencing pharmacists’ engagement in identification and response to DSVA (opportunity)

Pharmacies were perceived as highly accessible local health providers on the high street, with sexual health consultations offering an ideal ‘Opportunity’ to enquire about DSVA in a private consultation room. Pharmacists’ perception of external physical and psychological factors that can enable or hinder their engagement in DSVA work differed between standalone independent and corporate chain pharmacies. All participants saw the accessibility and inclusivity of the community pharmacy as enablers for DSVA work. They thought that the public perceived community pharmacy as local health provider on high street and a first point of call with common health problems. Extended opening hours meant that working people and clients from a wider area could attend. Walk-in health services meant increased accessibility compared to general practice with pre-booked appointments (Table 3, quote 1).

**Table 3:** External factors influencing pharmacists’ engagement in identification and response to domestic and sexual violence and abuse (Opportunity)

Quote number	Supporting quote	Participant
Quote 1	‘I think we could have a great role here [respond to domestic and sexual violence and abuse] because we’re accessible all the time. People feel quite comfortable coming in and we always have a room we can talk in, okay, confidentially. And also, we’re open. We are a hundred-hour pharmacy here, so we’ve got seven until eleven. I think it’s a good setting because I think it’s more accessible to people than doctors’ surgery is.’	Pharmacist 4, corporate chain pharmacy, South West
Quote 2	‘We’re a community pharmacy. We’ve been here since 1990, and we formed a very strong relationship with the local community, local schools, the elderly population, the church locally, and we’re quite a valued service in this area.’	Pharmacist 5, standalone independent pharmacy, London
Quote 3	‘I think we’re put in a good place to do that [respond to domestic and sexual violence and abuse] because I think pharmacy and pharmacists can do more in terms of the fact that I think the population still view us as a dispensing machine where they bring in a piece of paper, they get some tablets out of it which is a shame because we are able to do more than that.’	Pharmacist 6, corporate chain pharmacy, South West
Quote 4	‘sometimes they don’t want me involved, certain ladies, especially of certain ethnic populations, they might feel very shy and embarrassed, they will not discuss certain things with them. ……You find that with a lot of the Somalian and Islamic ladies: that they do not want to.... The way their culture is, and they’ve been told not to discuss certain things. So my ladies [female pharmacy assistants] are well trained in that.’	Pharmacist 5, male, standalone independent pharmacy, London

Participants from independent pharmacies described having worked within local communities for a long time and being ideologically committed to supporting the local communities. They felt they provided more personalised care, developed relationships with customers over time and offered continuity of contact and put the needs of the local people above profit (Table 3, quote 2). At the same time, they had fewer pharmacists who mainly worked at the back of the shop dispensing medication. Pharmacy assistants and technicians were the first point of contact who triaged clients’ enquiries. Participants from corporate chain pharmacies had more resources to free up pharmacists’ time for health services. They also thought that they offered clients more anonymous health services due to the bigger footfall and wider catchment area.

All pharmacies had consultation rooms, which they used as an environment enabling privacy, confidentiality and safety when providing consultations on sensitive topics (e.g. EHC). However, few participants from small independent pharmacies thought that even in the consultation room, some clients could feel too exposed because of the possibility of knowing and being known to the staff and people attending from the same community. One pharmacist said that despite offering consultation in a private room, some clients want conversation over the counter.

Pharmacists were concerned that public and other professionals are not yet ready to use pharmacies as providers of public health services including response to DSVA. Participants thought that most people still perceive pharmacies as medicine suppliers and attend for a transaction of medicine rather than for a healthcare consultation (Table 3, quote 3). Pharmacists suggested varied strategies to raise awareness in the community of the possibility for women approaching pharmacists about DSVA. These were mostly modelled on public health campaigns with posters for the window/inside the pharmacy and leaflets for people to take away. One pharmacist suggested that it would be important to advertise this new capacity outside the pharmacy in places where women would be likely to see the information.

Pharmacists serving multicultural communities identified language and cultural barriers to engaging with patients from minority ethnic groups. Female pharmacy assistants were key for facilitating communication between women seeking EHC and male pharmacists (Table 3, quote 4).

### Pharmacists’ motivation to identify and respond to DSVA (Motivation)

Pharmacists’ ‘Motivation’ to enquire about DSVA was driven by potential positive client outcomes and a desire be more involved in public heath interventions, but they needed organisation- and system-level support and financial remuneration. Pharmacists believed that identification and response to DSVA could be part of their role in provision of public health services but had concerns about their confidence and competence, negative consequences and lack of incentives for engaging in DSVA work. All pharmacists saw themselves as healthcare providers with limited expertise in diagnosing and managing clinical conditions. Participants valued extended pharmacy role comprising traditional medicines supply and provision of new public health services. Some pharmacists saw DSVA work as an opportunity to enhance existing health services for vulnerable populations. Pharmacists wanted to provide holistic healthcare and suggested integrating identification and response to DSVA into the existing commissioned EHC service, supervised consumption of opioid substitution therapy and needle and syringe exchange. Some saw potential for integrating DSVA work into medicine use reviews, undergraduate pharmacy courses and public health campaigns.

Participants from standalone independent pharmacies valued their social capital in the local community and believed that they could become an essential part of a multisectoral response to DSVA (Table 4, quote 1). All pharmacists felt motivated to get involved in the provision of more public health services and described the intention for constant professional development and positive examples from their own practice (Table 4, quote 2). However, pharmacists felt uncertain about their ability to fit DSVA work into the current workload. Participants from small independent pharmacies with one duty pharmacist worried that provision of more public health services can reduce their time for earning their ‘bread and butter’ by supplying medicines. In contrast, participants from corporate chain pharmacies described a trend towards employing greater numbers of supporting staff to free-up pharmacist time for providing more public health services. Chain pharmacies also had written safeguarding and referral policies and processes, while independent pharmacies relied on the responsible pharmacist and informal processes. Several participants had concerns about negative consequences of engaging in DSVA work such as lack of clinical supervision to offload emotional burden, client complaints, unclear insurance and liability for DSVA work.

**Table 4:** Pharmacists’ Motivation to identify and respond to domestic and sexual violence and abuse (Motivation)

Quote number	Supporting quote	Participant
Quote 1	‘Because we are here in the community. It’s like Eastenders [UK TV show], we get to know everything. We get to know a lot of gossip as well: people tell us. We don’t repeat anything, but information comes to us. So it’s good to know that if we are involved, and we can prevent something terrible happening, I think it’s always benefit to any community. I think as a community everybody has to work together, all health professionals. And you can’t have one health professional and not another, whether it’s nurses, community nurses, district nurses; if they’re in the houses; we should all be working together. Because we would be the eyes and the ears.’	Pharmacist 7, standalone independent pharmacy, London
Quote 2	‘Because dispensing’s quite boring [laughs], yeah, so this is the opportunity to actually speak with the patients and get to know the patient, and you’re actually utilising more of your skills that you’ve attained during university, and so I think it’s a good thing. We’d welcome more services actually.’	Pharmacist 1, standalone independent pharmacy, London
Quote 3	‘Yeah, if you have a commissioned service, then... When there’s money involved people become a bit more proactive normally, yeah, and then you feel that the time that you’re spending as well, you’re getting something in return as well. Although it shouldn’t be like that, but it does make people feel like that. So maybe you will see a bigger result if there was some sort of funding available.’	Pharmacist 1, standalone independent pharmacy, London

One third of pharmacists said that financial incentivization would facilitate their engagement in identification and response to DSVA. They explained that community pharmacy is a private business paid per medicine or health service supplied as opposite to general practice paid per population served. Although independent pharmacists highlighted their commitment to the needs of the local community over payment, most pharmacists thought that budget for DSVA work should be allocated the same way as for other commissioned public health services (Table 4, quote 3). Pharmacists had mixed opinions about how budgets should be allocated for DSVA work. One participant thought it would be difficult to derive an outcome-based payment. Other thought that paying for locum cover, so pharmacists could attend DSVA training would be sufficient. Participants suggested LPC as an intermediary for negotiating funding for DSVA work with the local health commissioners. An existing information system PharmOutcomes was described as the online platform for linking provision of public health services and payment, which could be used for recording and remunerating for DSVA work on a payment-by-activity basis.

## Discussion

### Main findings of this study

We explored barriers and facilitators to pharmacists’ engagement in identification and response to DSVA and mapped them on the COM-B model to inform pharmacy-specific adaptations of the IRIS GP intervention. Qualitative interviews with community pharmacists found that they have some Capacity, Motivation and see Opportunities for integrating identification and response to DSVA into existing commissioned public health services. The main facilitators for pharmacist engagement in DSVA work were expertise and experience in providing commissioned public health services (Capability), the accessibility and inclusivity of the pharmacy setting (Opportunities), perceived relevance of DSVA work to their role and intention to get more involved in public health services (Motivation). Barriers to engaging in DSVA work included: (i) uncertainty about how to identify and respond to clients affected by DSVA in the time available, lack of training, policy/guidance, judgemental attitudes (Capability), (ii) limited resources, societal perception of pharmacy role, language and cultural norms (Opportunities), (iii) fear of negative consequences, lack of support to offload emotional burden and lack of reward (Motivation).

Pharmacists suggested integrating DSVA identification and response into existing public health services, for example sexual health consultations. They saw LPCs and existing system of remuneration for commissioned public health services as vehicles for creating opportunities and motivation for engaging in DSVA work.

### What is already known on this topic

One systematic review found some evidence on the acceptability, feasibility and effectiveness of pharmacy-based public health interventions for smoking cessation and weight loss.^40^ The Cochrane review showed that pharmacy-based interventions can change behaviour of pharmacy staff and clients.^41^ Our systematic review^27^ and analysis of routine data^28^ found an association between exposure to DSVA and increased use of EHC. This supports the case for extending primary healthcare response to DSVA to community pharmacy supplying 50% of all EHC. UK community pharmacies are well placed to integrate DSVA interventions, as they are embedded in local communities and offer free walk-in public health services under PGD.^14^ Commissioned sexual health services are targeted at younger people who are more vulnerable to DSVA. Our scoping literature review identified no evaluations of pharmacy based DSVA interventions, including the recent codeword schemes in response to the COVID-19 pandemic. Two exploratory studies with US pharmacy professionals and clients found that there is a potential for pharmacy-based identification and response to DSVA.^42^ The studies of pharmacists’ and clients’ perspectives on providing public health services^43^^,^^44^ and exploratory studies on identifying and responding to DSVA in pharmacies^30^^,^^33^ reported similar barriers—limited Capability and Motivation among professionals and lack of external Opportunities. Research recommendations suggested theory-based development of interventions for behaviour change in pharmacy staff and clients.^41^

### What this study adds

We applied the COM-B model to identify barriers and facilitators to engaging pharmacists in identification and response to DSVA during provision of commissioned public health services. Pharmacists were confident and motivated to provide public health services but lacked Capability and Motivation for engaging in identification and response to DSVA.

Pharmacy setting offers Opportunities for integrating DSVA work in existing public health services. Pharmacists welcome training on DSVA, ongoing support, allocated funding for DSVA work and awareness raising campaign for the public on their extended public health role. LPC and PharmaOutcomes platform can be used to deliver the training and remunerate for DSVA work integrated into existing commissioned public health services. These findings will inform pharmacy specific adaptation of the IRIS GP programme through matching the identified barriers and facilitators with target behaviours on the Theoretical Domains Framework and using the Behaviour Change Wheel to choose behaviour change technics.^38^

Our findings are applicable to the recent codeword schemes in community pharmacies, which have not yet been formally evaluated. In line with the previous systematic reviews,^21^^,^^22^ our study theoretically informed by the COM-B model^38^ found that a one off training on DSVA is not enough for changing professional behaviour. While the training may have increased pharmacist’s ‘Capability’ to respond to DSVA, issues still arose in relation to ‘Opportunity’ to enquire about DSVA in a private consultation room and their ‘Motivation’ to do it. This suggests that community pharmacists need ongoing support at the organisation- and system-level and remuneration for every DSVA encounter to develop and sustain their ‘Capability’, external ‘Opportunities’ and ‘Motivation’ for engaging in the evidence-based health- systems response to DSVA.

### Limitations of this study

We did not interview pharmacy clients and therefore further research is required to examine their views on the acceptability of the DSVA work. This study took place before the UK Home Office introduced the codeword schemes in community pharmacies. Pharmacists’ ‘Capability’ and external ‘Opportunities’ could have changed because of the training and promotional materials provided as part of these schemes.^17^

## Authors’ contribution

JH, GF, NVL conceived and designed the study. NVL and TS conducted interviews. NVL, TS, and JH analysed the data and produced first draft. All authors contributed to five revisions of the manuscript. All authors read and approved the final manuscript.

## Data Availability

The data underlying this article will be shared on reasonable request to the corresponding author.

## Notes

**Natalia V. Lewis**, PhD, Research Fellow in Primary Care

**Tracey Stone**, PhD Medical Ethics, Senior Research Associate

**Gene S. Feder**, B.Sc., M.B.,B.S., M.D., F.R.C.G.P., Professor of Primary Care

**Jeremy Horwood**, B.Sc., PhD, Professor of Social Sciences and Applied Health Research
